# *Fusobacterium necrophorum* subsp. *necrophorum* Liver Abscess with Pylephlebitis: An Abdominal Variant of Lemierre's Syndrome

**DOI:** 10.1155/2020/9237267

**Published:** 2020-01-13

**Authors:** Natasa Radovanovic, Igor Dumic, Mladjen Veselinovic, Susanne Burger, Tamara Milovanovic, Charles W. Nordstrom, Eric Niendorf, Poornima Ramanan

**Affiliations:** ^1^Internal Medicine Residency Program, Icahn School of Medicine at Mount Sinai, New York City, NY, USA; ^2^Mayo Clinic Alix College of Medicine and Science, Rochester, MN, USA; ^3^Division of Hospital Medicine, Mayo Clinic Health System, Eau Claire, WI, USA; ^4^Infectious Disease Fellowship Program, University of Utah, Salt Lake City, UT, USA; ^5^Division of Infectious Disease, Albert Einstein College of Medicine, Bronx, New York City, NY, USA; ^6^Department of Gastroenterology and Hepatology, Clinical Center of Serbia, Belgrade, Serbia; ^7^Division of Radiology, Mayo Clinic Health System, Eau Claire, WI, USA; ^8^Division of Infectious Disease, University of Colorado, Denver, CO, USA

## Abstract

Liver abscess associated with suppurative portal vein thrombosis (pylephlebitis) secondary to *Fusobacterium necrophorum* has been rarely reported. It is considered to be an abdominal variant of Lemierre's syndrome associated with significant morbidity and mortality. We report a case of 69-year-old man who developed liver abscess and pylephlebitis due to *F. necrophorum* with an unclear source of infection. We discuss the pathogenesis, diagnosis, and treatment strategy for this entity, with a review of previously published cases of pyelephlebitis due to *F. necrophorum* in regard to their clinical presentation and outcome.

## 1. Introduction

*Fusobacterium necrophorum* is a non-spore forming, obligate anaerobic, Gram-negative bacillus that is part of the normal flora of human oropharynx, gastrointestinal, and urogenital tracts [1]. Human infections are usually caused by *F. necrophorum* subsp *funduliforme*, while infections by *F. necrophorum* subsp *necrophorum* are more commonly seen in animals [1]. *F. necrophorum* is unique among non-spore forming anaerobes for its ability to cause severe infection and the fact that infection can be acquired exogenously [1]. These bacteria are usually associated with necrobacillosis—a rare but severe, potentially life-threatening necrotizing infection. Lemierre's syndrome is a classic example of human necrobacillosis wherein acute primary infection of oropharynx is followed by secondary septic thrombophlebitis of internal jugular vein and subsequent septic embolization at various sites [2]. Pylephlebitis (suppurative, infected portal vein thrombosis (PVT)) is a rare but serious complication of intra-abdominal or pelvic infection and is associated with significant morbidity and mortality [3, 4]. Pylephlebitis due to *F. necrophorum*, also known as abdominal variant of Lemierre's syndrome, has been rarely reported.

## 2. Case

A 69-year-old man was seen in his primary care office during summer complaining of profound fatigue, fever, and right upper quadrant (RUQ) abdominal pain. His symptoms have been intermittent, occurring without a particular pattern over the last two months. With time, the intensity and frequency of his symptoms had increased which prompted him to seek medical evaluation. His abdominal pain was intermittent, dull, and nonradiating, without prandial or positional component. Medical history was significant for mild intermittent asthma, benign prostatic hyperplasia, and squamous cell carcinoma of the tongue for which he underwent partial tongue resection followed by radiation one month prior to the onset of current symptoms. He was a former smoker and did not drink alcohol or use illicit drugs or over the counter medications. He lived in the Midwestern United States and had not traveled outside the country. He denied having any pets or contact with animals. Family history was negative for inherited hypercoagulable disorders or cancer. His age appropriate cancer screening was up to date, and he was considered cured from his locally invasive tongue cancer.

**Figure 1:**
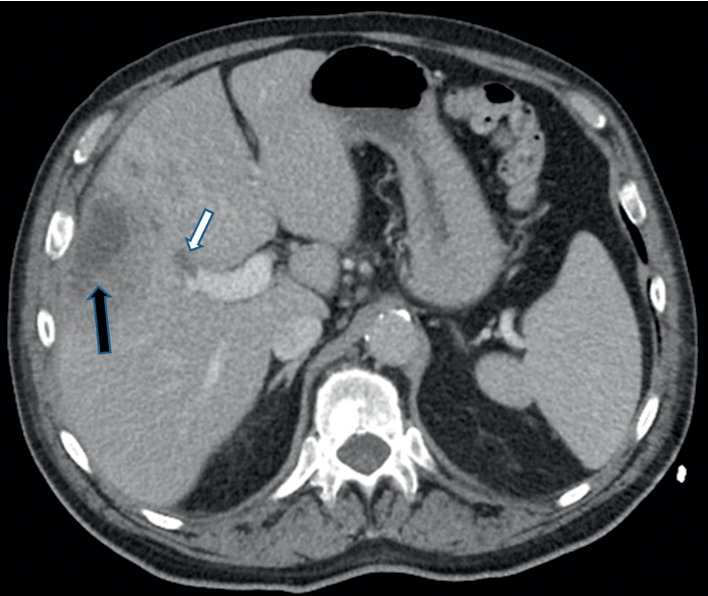
Contrast-enhanced axial CT image of the upper abdomen demonstrating the liver abscess (black arrow) and thrombus in the portal vein (white arrow).

In the office, he was found to have fever of 39°C, a heart rate of 102 beats per minute, a blood pressure of 148/81 mmHg, and normal respiratory rate and oxygen saturation. Physical exam was remarkable for well-developed, ill-appearing man in no acute distress. There was no cervical lymphadenopathy or mouth ulcer, and the tongue resection site appeared well healed without erythema or exudate. The teeth appeared healthy without caries or gingivitis. Tonsils appeared healthy and without erythema or exudate. The lungs were clear to auscultation bilaterally; heart sounds were regular and without murmurs. His abdominal exam was significant for right upper quadrant (RUQ) tenderness on deep palpation without signs of peritonitis, and bowel sounds were normoactive. He had no skin rashes, telangiectasia, or icterus. Neurological exam was nonfocal. Complete blood cell count (CBC) showed neutrophilic leukocytosis (white blood cell count—17.6 × 10^9^/L with 90 percent neutrophils). Liver function tests (LFT) were within normal range with the exception of alkaline phosphatase (ALP) which was 300 ng/dl (120 ng/dl upper limit normal). He was admitted for further evaluation and management of sepsis. Upon admission he received intravenous (IV) fluids and was started on ceftriaxone and metronidazole intravenously after blood cultures were drawn. Following IV fluid administration and antibiotic therapy his vital signs normalized. Further workup for RUQ abdominal pain was carried out. It initially included abdominal ultrasound which was read as normal. Contrast-enhanced computed tomography (CT) scan of abdomen was done and revealed an ill-defined mass in the right liver lobe concerning for abscess and thrombosis of the portal vein (Figure 1). The spleen size was measured 10.5 cm vertically in its longest dimension, and there were no collaterals seen within splanchnic veins. Magnetic resonance imaging (MRI) of the abdomen was obtained to further delineate characteristics of the mass, and this showed multifocal masses in the right liver lobe with associated satellite masses concerning for malignancy. The largest liver lesion was biopsied which yielded purulent and hemorrhagic material. Pathology of the aspirate was negative for malignancy and showed necrotic debris with abundant neutrophils. After 36 hours, both sets of anaerobic blood culture bottles grew thin, filamentous Gram-negative bacilli. They were later identified to be *F. necrophorum* subsp. *necrophorum* by matrix-assisted laser desorption ionization-time of flight (MALDI-TOF). The liver abscess cultures also grew the same organism. Further workup for pylephlebitis included testing for prothrombic disorders, occult malignancy, and cirrhosis (most common causes associated with development of PVT) (Table 1). Workup for *F. necrophorum* liver abscess and bacteremia was aimed to identify the primary source of infection such as oropharyngeal infection, otitis media, mastoiditis, and abdominal or pelvic infection. Detailed physical exam and imaging (contrast-enhanced CT head and neck, abdomen, and pelvis) of these anatomical compartments was negative for the source of bacteremia. The liver abscesses were drained and intrahepatic stents were placed with gradual decrease in the size of the abscess. He completed three weeks of ceftriaxone and metronidazole therapy and was transitioned to an additional two weeks of oral therapy with amoxicillin-clavulanic acid. He was also anticoagulated with enoxaparin and bridged to warfarin to achieve an international normalized ratio (INR) between 2 and 3 with a plan to continue anticoagulation for either three months or until resolution of PVT on repeat CT scan, whichever was longer. One month following discharge, he was seen in primary care office and was doing well, and his symptoms had resolved. He was lost to subsequent follow-up.

## 3. Discussion

Noncirrhotic, nonmalignant PVT is rare and is usually associated with infection, trauma, or prothrombic disorder. In about 25 percent of cases, the etiology remains elusive despite extensive workup [3]. Pylephlebitis (suppurative thrombophlebitis of the portal vein) is a rare complication of intra-abdominal or pelvic infection [4]. Choudhry and coauthors in their retrospective analysis identified 95 patients who developed PVT following an intra-abdominal infectious process over a period of 10 years at Mayo Clinic [5]. In their study, pylephlebitis was most commonly associated with pancreatitis (31%) and diverticulitis (19%). A systematic review of the literature by Kanellopoulou et al. [6] identified diverticulitis and appendicitis to be the most common intra-abdominal infection leading to pylephlebitis. In both studies, polymicrobial infection was commonly identified, followed by monomicrobial infection with *Streptococcus viridians*, *Bacteroides fragilis*, and *Escherichia coli.* The rates of bacteremia were 44% and 42% in these two studies, respectively [5, 6]. Mortality rates in these studies were 11% and 19%, respectively [5, 6]. Neither of the studies documented *F. necrophorum* as a causative agent. Liver abscess as an intra-abdominal source of infection in this study [5] was found to be associated with PVT in only 2% of cases.

We searched the PubMed database for articles published in English using the following key words alone or in combination: pylephlebitis, suppurative portal vein thrombosis, *Fusobacterium necrophorum*, necrobacillosis, septic portal vein thrombosis, and infected portal vein thrombosis. Our search yielded 6 cases [7–12] (Table 2). The median age of patients was 37 years (ranging from 19 to 53 years) with 5:1 male to female ratio. It is to be noted that although our patient was male, he was older. Similar to classic Lemierre's syndrome, the majority of patients in our literature search were young, healthy, and without significant comorbidities including malignancy, liver cirrhosis, or inherited or acquired prothrombic disorder. Interestingly, three out of four cases that reported patient's social habits report heavy alcohol use. In our systematic review all infections were monomicrobial and were community acquired.

The source of infection was confirmed to be from gastrointestinal source in one-third of patients. In one-third, the source was presumed (gastrointestinal or urogenital system), and in one-third, the source was not identified. We believe that in our patient, pyelephlebitis was secondary to liver abscess. This is similar to another case report [10] where *F. necrophorum* liver abscess was associated with pyelephlebitis. Interestingly, in that case, the patient also had pancreatitis which is a well recognized and common cause of PVT. Despite an extensive and robust search for an underlying source of bacteremia that would have led to development of liver abscess and subsequent suppurative PVT infection, we were unable to identify one in our patient. He had a well-healed scar from his tongue resection; there was no mucosal damage, nor signs and symptoms of tonsillitis, gingivitis, otitis media, or mucositis. Abdominal CT scan was negative for intra-abdominal malignancy or other source of infection, and we excluded other causes of portal vein thrombosis (Table 1). Hence, we firmly believe that this was indeed thrombosis caused by infection—pylephlebitis.

*F. necrophorum* is an exceedingly rare cause of pyogenic liver abscess [13]. Similar to pyleophlebitis, fusobacterial liver abscess is more common in males upon a recent literature review [13], describing 41 of 48 cases occurring in men. Similar to our patient, 8 of these 48 cases were also cryptogenic, without a source of infection identified. In this review, the majority of *F. necrophorum* liver abscesses (20/23) were monomicrobial with only 3 of 23 having isolated a second pathogen (polymicrobial). This review found that unlike other pyogenic liver abscesses, those caused by fusobacterium spp usually lack traditional risk factors such as malignancy, dialysis treatment, immunosuppression, and older age [13]. Our patient had slightly higher risk for liver abscess given his older age and recent therapy for malignancy.

The propensity of *F. necrophorum* to cause thrombosis is explained by its ability to induce platelet aggregation [14]. In addition to internal jugular and portal vein, other sites such as cavernous sinus [15] and cerebral venous thrombosis [16] both secondary to *Fusobacterium* meningitis have been described in the literature.

Unlike liver abscesses that are easily discovered on routine imaging, clinical diagnosis of pylephlebitis is challenging; however, all reported cases had a triad of dull RUQ or epigastric pain, fever, and leukocytosis. The most common liver enzyme abnormality was ALP (75% of cases) (Table 2). The diagnosis of septic PVT requires the demonstration of thrombus formation in portal vein or its branches in the setting of positive blood cultures [17].

Pylephlebitis is treated with a combination of antibiotics and anticoagulation [17]. Antibiotic classes that cover anaerobes including *F. necrophorum* are a combination of beta-lactam and beta-lactamase inhibitor (e.g. piperacillin-tazobactam), metronidazole, ceftriaxone, clindamycin, and carbapenems. Duration of treatment in reported cases was highly variable ranging from four to seven weeks. Prolonged duration of therapy is recommended [18]. In cases of liver abscess, source control and abscess drainage are recommended in addition to antimicrobial therapy [13]. Furthermore, abscess aspirate is useful to confirm etiology and guide an appropriate antimicrobial regimen. Most regimens included initial two to three weeks of IV therapy followed by oral therapy. Anticoagulation was pursued in five out of six reported patients. Warfarin was the most commonly used anticoagulant, and duration of therapy was variable from three to six months. Direct-acting oral anticoagulants (DOACs) were not used in cases reported thus far. The benefit of anticoagulation therapy has been debatable [18]. Due to the rarity of this disorder clinical trials are difficult to conduct, and recommendations are based on case report and expert opinion. Given limited data on the benefit of anticoagulation in this setting, we recommend careful analysis of potential risks and benefits on a case-by-case basis prior to starting anticoagulation [19]. While some authors suggest universal use of anticoagulation due to higher recanalization rates [6, 20], others recommend selective use of anticoagulation for pylephlebitis in the following scenarios: documented progression of thrombus while on antibiotics, fever unresponsive to treatment, and the presence of a hypercoagulable state [21]. In chronic PVT, anticoagulation can prove more challenging as the majority of the patients have varices with an associated bleeding risk; however, patients with acute nonmalignant and noncirrhotic PVT (including pylephlebitis) are usually considered safe to be anticoagulated with low bleeding rates [22]. It should also be noted that patients with PVT in the setting of ascites and associated splenic vein thrombosis have lower recanalization rates [23].

Earlier studies reported mortality from pylephlebitis to be as high as 35% [17]. It was attributed mainly to delay in diagnosis and antibiotic administration causing overwhelming sepsis. Of note, mortality rate was lower in two recent studies (11 to 19%) [5, 6] and was zero among the 6 reported cases with pylephlebitis due to *F. necrophorum* (Table 2), likely reflecting earlier recognition of pylephlebitis due to advances in diagnostic imaging leading to timely initiation of antibiotics.

Limitations of this case include the fact that the patient was lost to follow-up. This precluded our ability to confirm the resolution of infection or PVT. Additionally, we did not test for Prothrombin G20210A (Factor II) mutation; therefore, hypercoagulability secondary to this could not be excluded completely.

## 4. Conclusion

Despite its rare occurrence, *F. necrophorum* pylephlebitis is a potentially serious complication of *Fusobacterium* bacteremia. Clinicians should keep this entity in mind in patients who present with triad of abdominal pain, fever, and leukocytosis without other obvious intra-abdominal infection.

## Figures and Tables

**Table 1:** Summary of extensive workup that was done to investigate etiology of portal vein thrombosis in our patient. It includes three disease categories that are most commonly associated with development of PVT. All listed laboratory tests were either negative or the values were within normal limits.

Prothrombic disorders (inherited and acquired)	Occult malignancy	Cirrhosis
JAK 2 V617 F mutation Fibrinogen Thrombin time Protein C levels^*∗*^ Protein S levels^*∗*^ Factor V Leiden mutation ANA PTT Anti-*β*2 glycoprotein antibodies Antithrombin functional activity^*∗*^ Lupus anticoagulant Anticariolipin antibodies	CEA CA-19- 9 AFP PSA Abdominal CT scan Abdominal MRI	Platelets INR Albumin Total bilirubin Abdominal US Abdominal CT scan Abdominal MRI HBV antigen and antibodies HCV antibodies

AFP, alfa-fetoprotein; ANA, anti-nuclear antibodies; CA, carbohydrate antigen; CEA, carcinoembryonic antigen; CT, computed tomography; HBC, hepatitis B virus; HCV, hepatitis C virus; INR, international normalized ratio; MRI, magnetic resonance imaging; PSA, prostate specific antigen; PTT, partial thromboplastin time; US, ultrasound. ^*∗*^The values of these tests might not be accurate during the acute phase of thrombosis.

**Table 2:** Summary of the patient demographics, underlying comorbidities, clinical features, treatment, and outcomes of all previously published case reports of patients with septic portal vein thrombosis secondary to *Fusobacterium necrophorum*.

Case	Reference (year)	Country	Age	Sex	Primary source of infection	CIR	CA	ALC	ABP	HCD	Fever	Leukocytosis	BCT	ASC	AST	ALT	ALP	Additional thrombosis	ABX (duration in weeks)	AC (duration in months)	Outcome
**1.**	Soo (1999)	Australia	31	M	GI tract (presumed)	No	NR	NR	Yes	No	Yes	Yes	Yes	No	79	133	295	SMV	CIP + MET (1) Augmentin (6)	Warfarin (6)	Full recovery
**2.**	Clarke (2003)	UK	19	F	GYN procedure or sore throat	No	NR	NR	Yes	n/a	Yes	Yes	Yes	Yes	NR	52	331	SMV	Abscess drainage CIP + MET + PCN (6.5)	Warfarin (long term)	Residual pH
**3.**	Redford (2005)	UK	53	M	NI	No	No	Yes	Yes	NR	Yes	Yes	Yes	NR	75	38	194	No	PCN + MET (2) CLINDA PO (5)	Warfarin (3)	Full recovery
**4.**	Shahani (2011)	USA	34	M	Pancreatitis liver abscess	No	NR	Yes	Yes	No	No	Yes	No	Yes	WNL	WNL	WNL	SMV + SV	VANC + MET + TIG (4)	No	Improved
**5.**	DePetrillo (2014)	USA	53	M	NI	No	NR	Yes	Yes	NR	Yes	Yes	Yes	NR	NR	70	152	No	Ertapenem (4)	Warfarin	Improved
**6.**	Akhrass (2015)	USA	32	M	Appendicitis	No	n/a	No	Yes	No	Yes	Yes	Yes	NR	WNL	WNL	WNL	SMV + SV	VANC + PIP-TAZ + MET + CLIND PO	THET + warfarin (6)	Full recovery
**7.**	Radovanovic (2018), current case	USA	69	M	NI oral cavity (presumed)	No	Tongue SCC	Yes	Yes	No	Yes	Yes	Yes	Yes	39	68	518	HV	Abscess drainage + CEFT + MET (3) + augmentin (2)	Warfarin (3)	Clinically improved (lost in follow-up)

ABP, abdominal pain; ABX, antibiotics; AC, anticoagulation; ALK, alcoholism; ASC, cultures from aspirate from intra-abdominal infection; BCT, blood cultures; CIR, cirrhosis; CIP, ciprofloxacin; CLINDA, clindamycin; F, female; GI, gastrointestinal; GYN, gynecological; HCD, hypercoagulable disorder; HV, hepatic vein; M, male; MET, metronidazole; NI, not identified; NR, not reported; PCN, penicillin; PH, portal hypertension; PIP-TAZ, piperacillin-tazobactam; PO, oral; SC, splenic vein; SVT, superior mesenteric vein; TIG, tigecycline; THET, trans-hepatic endovascular thrombolysis; UK, United Kingdom; USA, United States of America; VANC, vancomycin; WNL, within normal limits.
